# Classifying muscle parameters with artificial neural networks and simulated lateral pinch data

**DOI:** 10.1371/journal.pone.0255103

**Published:** 2021-09-02

**Authors:** Kalyn M. Kearney, Joel B. Harley, Jennifer A. Nichols

**Affiliations:** 1 J. Crayton Pruitt Family Department of Biomedical Engineering, University of Florida, Gainesville, Florida, United States of America; 2 Department of Electrical and Computer Engineering, University of Florida, Gainesville, Florida, United States of America; North Carolina State University, UNITED STATES

## Abstract

**Objective:**

Hill-type muscle models are widely employed in simulations of human movement. Yet, the parameters underlying these models are difficult or impossible to measure *in vivo*. Prior studies demonstrate that Hill-type muscle parameters are encoded within dynamometric data. But, a generalizable approach for estimating these parameters from dynamometric data has not been realized. We aimed to leverage musculoskeletal models and artificial neural networks to classify one Hill-type muscle parameter (maximum isometric force) from easily measurable dynamometric data (simulated lateral pinch force). We tested two neural networks (feedforward and long short-term memory) to identify if accounting for dynamic behavior improved accuracy.

**Methods:**

We generated four datasets via forward dynamics, each with increasing complexity from adjustments to more muscles. Simulations were grouped and evaluated to show how varying the maximum isometric force of thumb muscles affects lateral pinch force. Both neural networks classified these groups from lateral pinch force alone.

**Results:**

Both neural networks achieved accuracies above 80% for datasets which varied only the *flexor pollicis longus* and/or the *abductor pollicis longus*. The inclusion of muscles with redundant functions dropped model accuracies to below 30%. While both neural networks were consistently more accurate than random guess, the long short-term memory model was not consistently more accurate than the feedforward model.

**Conclusion:**

Our investigations demonstrate that artificial neural networks provide an inexpensive, data-driven approach for approximating Hill-type muscle-tendon parameters from easily measurable data. However, muscles of redundant function or of little impact to force production make parameter classification more challenging.

## Introduction

Accurately simulating human movement requires the appropriate selection of muscle-tendon parameters. These parameters define the modeled muscle-tendon actuators that mathematically transform neural excitations into muscle forces, thereby enabling joint movement. Hill-type muscle models [1, 2] are the most widely used muscle-tendon actuator and consist of a contractile element, series elastic element, and parallel elastic element to represent the force-length and force-velocity properties of muscle. Five parameters define Hill-type models: maximum isometric force, optimal fiber length, tendon slack length, pennation angle, and maximum contraction velocity of the muscle-tendon unit [3].

The five Hill-type muscle-tendon parameters are difficult or impossible to measure *in vivo*, and the need to estimate these parameters compromises musculoskeletal model accuracy. For example, generic parameter values are typically estimated from cadaveric specimens [4–7] or animals [8, 9]. To improve accuracy, most studies leverage anthropometric scaling factors, meaning muscle-tendon parameters are adjusted based on a subject’s height and/or weight [10, 11]. However, anthropometric scaling does not capture known parameter variation due to age [12, 13], sex [14], physical training [15], or muscle function [16]. To overcome this limitation, others have employed medical imaging technologies, such as magnetic resonance imaging and ultrasound, to estimate Hill-type parameters [17]. These approaches can inform selection of subject-specific parameters; however, they can be costly in terms of both time and money. Additionally, only pennation angle can be directly measured, meaning the other Hill-type parameters must be estimated from measurable anatomical corollaries (e.g., optimal fiber length estimated from measured fascicle length). A natural extension of this work is to estimate muscle-tendon parameters from easily measured data. For example, Garner and Pandy [18] proposed an optimization method for estimating three of the muscle parameters (tendon slack length, maximum isometric force, and optimal fiber length) from joint torque profiles. However, De Groote et al. [19] demonstrated that to what extent muscle parameter information is encoded within torque data varies based on the joint(s) studied and the limb position during torque testing. Thus, a generalizable approach for estimating muscle parameters from dynamometric data has not yet been realized.

Here, we examine to what extent artificial neural networks can be used to predict underlying muscle parameters from easily measurable datasets. Artificial neural networks are a machine learning method and have multiple advantages making them attractive for complex biomechanical analyses. First, artificial neural networks can approximate complex nonlinear mappings, which are common to the musculoskeletal system. One example of this nonlinearity is displayed by the work of Pearlman et al. [20], which identified thumb-tip force to be a nonlinear function of muscle force in cadaveric specimens. Second, artificial neural networks can infer unseen relationships, such as the mapping between Hill-type muscle parameters and the joint movements they influence. Lastly, after training, artificial neural networks are computationally efficient and rapidly perform time-series classification [21], which can enhance analysis of dynamic data (e.g., joint angle trajectories, joint torques, and/or external forces versus time).

To examine the utility of artificial neural networks in the context of muscle parameter estimation, we introduce a simulation and machine learning framework to predictively model the relation between lateral pinch force and one key Hill-type muscle parameter: maximum isometric force. We selected lateral pinch data as our testbed, as it is an important activity of daily living [22] that is regulary characterized through easily measureable dynamometric data [23–25]. We specifically investigated whether neural networks could perform binary classification of the maximum isometric force of thumb muscles, which is analogous to identifying whether a muscle is stronger or weaker than average. Binary classification is a critical first step toward more complex machine learning models to identify continuous variables. For this work, we first generated and analyzed forward dynamic simulations of lateral pinch with varying maximum isometric force values for four thumb muscle actuators. This analysis confirmed that changes in maximum isometric force substantially impact thumb-tip force. We then evaluated the performance of two artificial neural network models (*feedforward* and *long short-term memory*) in classifying the maximum isometric force of thumb muscles. Feedforward neural networks are relatively simple, lacking “memory” as signals only travel from input to output [26]. Long short-term memory (LSTM) neural network models account for the dynamic behavior of the input data [27]. Each neural network model was evaluated via its test losses and accuracies in the analysis of increasingly complex datasets. Specifically, dataset complexity was varied by incorporating changes to varying numbers of thumb muscle actuators. We hypothesized that the LSTM model would classify maximum isometric force with greater accuracy than the feedforward model, but both models would decrease in accuracy as dataset complexity increased. Although we evaluate the proposed framework for predicting maximum isometric force of muscles during of lateral pinch, the described methods provide a framework for developing predictive models of any of the five Hill-type muscle-tendon parameters in various biomechanical systems.

## Methods

To examine the impact of varying the maximum isometric force of thumb muscles, we generated four datasets of lateral pinch. Dynamic lateral pinch simulations were produced via forward dynamics in OpenSim v. 4.1 [28]. Feedforward and LSTM neural network models were tested to predict the maximum isometric force of the muscle actuators varied using only dynamic thumb-tip force. The performance of each neural network model was quantified as test losses and accuracies using a 5-fold cross-validation process. We evaluated overall performance in predicting maximum isometric force as well as relative performance between the two neural network models.

### Lateral pinch datasets

**Fig 1:**
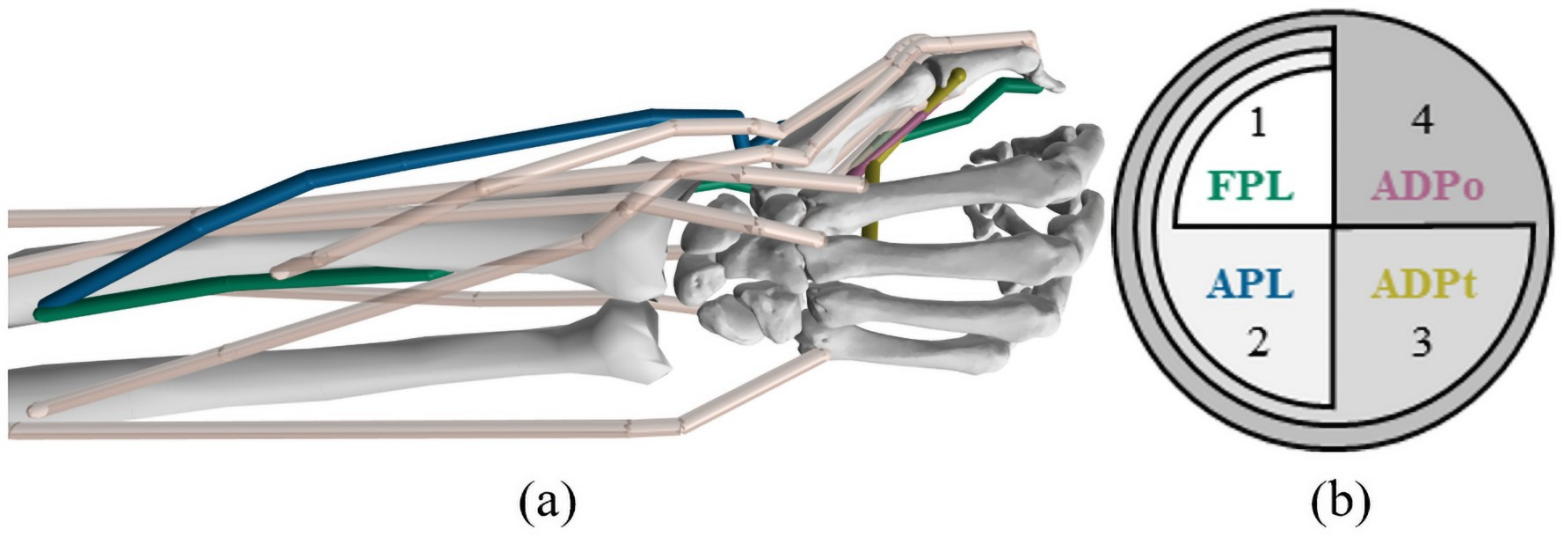
Musculoskeletal model and datasets. (a) Lateral pinch model used to generate dynamic thumb-tip force datasets [29]. (b) Values 1 through 4 correspond to Datasets 1 through 4, which varied the thumb muscle actuators shown. Datasets 1 through 4 contained 120, 1024, 2197, and 4096 simulations, respectively, representing different combinations of maximum isometric force values. For example, Dataset 1 included 120 variations to the FPL, while Dataset 4 included 8 variations to each the FPL, APL, ADPt, and ADPo.

Each simulation was processed using a previously developed musculoskeletal model of the wrist and thumb (Fig 1a) [29]. This musculoskeletal model includes fourteen muscle-tendon actuators (five wrist muscles, four extrinsic, and five intrinsic thumb muscles) and six degrees of freedom (two at the wrist, four across the thumb joints). Thumb-tip force was calculated at a point constraint located at the distal phalanx of the thumb, in a manner similar to Nichols et al. [29].

Each simulation varied the maximum isometric force input for thumb muscle actuators [the *flexor pollicis longus* (FPL), *abductor pollicis longus* (APL), transverse head of the *adductor pollicis* (ADPt), and the oblique head of the *adductor pollicis* (ADPo)]. We selected these muscle actuators as they contribute substantially to lateral pinch thumb-tip force [20]. To produce a wide range of physiologically relevant simulations, we calculated a range of maximum isometric force values (Table 1) using previously published measurements from *in vivo* and *in vitro* studies [30–32]. We estimated bounds by scaling the peak force range, calculated using a specific tension of 50.8 N/cm^2^ [30] and the physiological cross-sectional area [32, 33].

**Table 1:** Range of maximum isometric force values input into the lateral pinch model for each thumb muscle varied.

Muscle	Lower Bound ^a^	Mean	Upper Bound ^a^
FPL	63.52	201.00	338.48
APL	34.31	116.70	199.09
ADPt	3.11	59.90	116.69
ADPo	0.00^b^	102.1	220.54

^a^Bounds (representing 2 standard deviations from the mean) were estimated by scaling from the peak force range using previously published specific tension [30] and physiological cross-sectional area [32, 33] values.

^*b*^Two standard deviations below the mean maximum isometric force of the ADPo is exactly -16.34 N. This muscle’s lower bound was adjusted to meet physical constraints.

**Fig 2:**
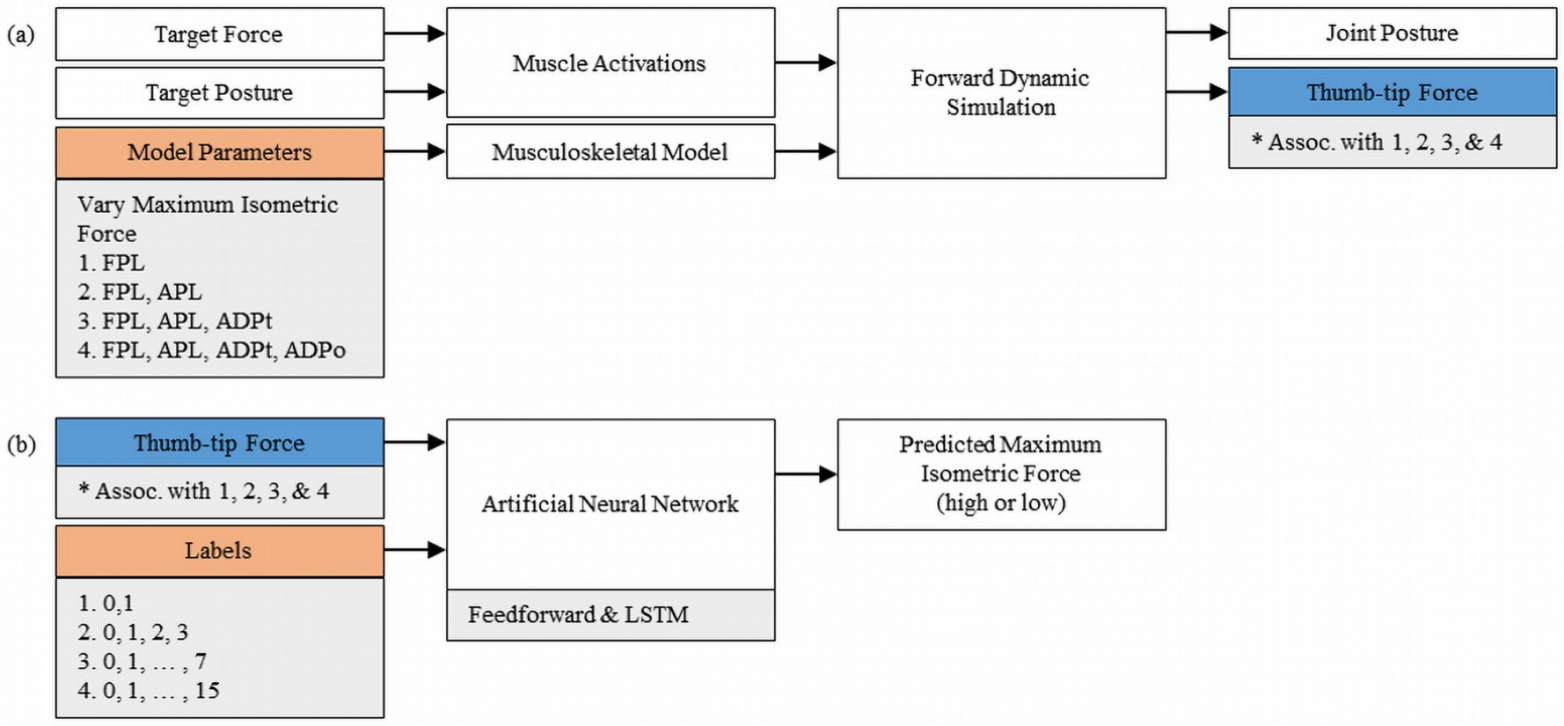
Simulation and machine learning workflow. (a) Workflow for producing simulated lateral pinch data. Muscle activations were attained via computed muscle control. (b) Workflow for training and testing artificial neural networks on simulated thumb-tip forces and time.

To enable isolated variation of the maximum isometric force of thumb muscles, we used forward dynamics to simulate lateral pinch (Fig 2a). Muscle activations calculated via computed muscle control served as inputs to the forward dynamic simulations. These activations were calculated to produce increasing thumb-tip force from 0 to 36.4 N magnitude (35 N in the palmar direction and 10 N in the ulnar direction) across 1.5 seconds while maintaining a target thumb posture (-15° carpometacarpal flexion, -20° carpometacarpal abduction, 20° metacarpophalangeal flexion, 40° interphalangeal flexion). The described target forces are both sufficient for many activities of daily living [22] and activated the muscles of interest. These activations were held constant across all simulations; thus, observed changes in output represent isolated changes in maximum isometric muscle force. The output of each forward dynamic simulation was a time-varying, three-component vector describing dynamic changes in thumb-tip force.

We generated four datasets (Fig 1b) to examine the impact of varying the maximum isometric force input of thumb muscles. Each dataset increased in complexity by including changes to the maximum isometric force for an increasing number of muscles. We first altered the maximum isometric force of the FPL (Dataset 1) and then added variations in APL (Dataset 2), ADPt (Dataset 3), and ADPo (Dataset 4) in order. This means that Dataset 1 varied only one muscle, whereas Dataset 4 included variations across all four. The FPL and APL were varied first as these muscles contribute the most to lateral pinch thumb-tip force [20] and importantly contribute to different directional components of thumb-tip force (the FPL flexes, the APL abducts). Within each dataset, the maximum isometric force values used for each muscle were uniformly sampled within the approximated range (Table 1). Dataset 1 included 120 simulations corresponding to uniform sampling across the full range of maximum isometric force values of the FPL. Datasets 2, 3, and 4 included 1024, 2197, and 4096 simulations, respectively. Importantly, these datasets scale substantially in size as needed to adequately train both neural network models for use in increasingly complex classifications. With the exception of Dataset 1, where the size was selected to not over-sample the isometric force space, dataset sizes were selected to be approximately double for each sequential dataset, while still uniformly sampling the maximum isometric forces for each muscle. For example, Dataset 4 incorporated 4096 simulations corresponding to all combinations of 8 maximum isometric force values for each of the four muscles (i.e., all combinations of maximum isometric force values = 84 or 4096 simulations).

All four simulation datasets were prepared to train and test both neural network models. To prevent neural network model bias favoring simulations of longer length, each simulation was interpolated to contain exactly 1000 time instances (i.e. 667 Hz). All simulations within a dataset were then grouped and labeled by whether the varied muscles were above (“high”) or below (“low”) the mean maximum isometric force values. This labeling reflects each neural network model’s task of predicting the maximum isomtric force of each muscle during lateral pinch. The modification of more muscles resulted in more labeled groups, and therefore 2, 4, 8, or 16 groupings of maximum isometric force value combinations in Datasets 1 through 4, respectively. To prevent neural network model bias, simulations were randomly assigned to training and testing sets; these sets defined the folds used during 5-fold cross validation.

### Neural network architecture

To inform how to predict maximum isometric force from minimal data, we employed feedforward and LSTM neural network models (Fig 2b). For each dataset, each neural network model classified the maximum isometric force of the altered thumb muscle(s) using only the dynamic thumb-tip force. Comparing the performance of the feedforward and LSTM models informed how muscle contraction dynamics may be leveraged to predict muscle parameters. Where the feedforward model treats each simulation time as independent, the LSTM model treats each simulation time sequentially. The LSTM model effectively uses the behavior of force data across time to inform its predictions.

Both neural network models were similar in structure, containing 4 input nodes (the three-dimensional force vector and time), 4 hidden nodes, and 2, 4, 8, or 16 output nodes (one “high” or one “low” node for each muscle) for Datasets 1 through 4, respectively. Three-component thumb-tip force vectors were selected as they are easily measurable *in vivo* and directly impacted by the modifications to the maximum isometric force of muscles of the thumb. Each neural network employs a sigmoid activation function, an Adam learning rate optimizer [34], and contains the hidden nodes in a single hidden layer. The output nodes correspond to the labeled groups of each dataset, and therefore increased in number to encompass the groupings required to classify the maximum isometric force of additional muscles.

The learning rate has a substantial impact on neural network model performance; thus, we adjusted this value for the analysis of each dataset for both neural network models. During learning rate tuning, training terminated when either the losses did not decrease for 25 consecutive epochs or the network trained for 100 epochs. These termination criteria were selected to provide enough epochs for convergence of weights while halting neural network model training for those that were not converging. We tested learning rates between 10^−7^ to 1 and selected the final learning rate via the criteria of minimizing losses [35]. The final learning rates selected for the analysis of each dataset are provided in Table 2. Training and testing for each learning rate were conducted in conjunction with 5-fold cross-validation. This approach reduced overfitting of the neural network to the training data.

**Table 2:** Muscles varied, training simulations, and final learning rates for each neural network model across datasets.

Dataset	Muscle(s) Varied	Training Simulations ^a^	Learning Rate *Feedforward*	Learning Rate *LSTM*
1	FPL	96	7.67 × 10^−5^	9.12 × 10^−5^
2	FPL, APL	819	2.53 × 10^−6^	9.86 × 10^−6^
3	FPL, APL, ADPt	1758	3.61 × 10^−6^	8.38 × 10^−6^
4	FPL, APL, ADPt, ADPo	3277	5.00 × 10^−7^	8.00 × 10^−7^

^a^Each simulation was a 1000 (simulated instances) by 4 (time and 3-component force vector) matrix.

### Analysis

Predicting maximum isometric force from thumb-tip force is only feasible if this parameter considerably influences thumb-tip force. We therefore first examined how varying maximum isometric force of thumb muscle actuators affects thumb-tip force. For this analysis, we calculated the means and standard deviations of peak thumb-tip forces across all simulations within each labeled group. This provided mean distributions of force vectors in 3D space for each unique simulation scenario. Within each dataset, we then calculated the percent volume overlap between these distributions. No volume overlap indicates that the simulated values of maximum isometric force uniquely map to the produced thumb-tip forces, while high overlap indicates that this mapping is not unique. Thus, the thumb-tip force distributions and percent overlap reveal to what extent thumb-tip force is sensitive to alterations in the maximum isometric forces of thumb muscles. Furthermore, the overlap in thumb-tip force across labeled groups reveals possible sources of classification error for each neural network model.

We also analyzed the distribution of test accuracies and losses associated with the final neural network models as evaluated via 5-fold cross-validation. For a well-performing model, accuracies should generally increase and losses should decrease across epochs until stabilizing at their final values. Training was terminated after 100 epochs, as the losses and accuracies were stable, indicating solution convergence. We calculated 95% confidence intervals for the test accuracies and losses for each neural network model across epochs. To test whether accounting for the dynamic behavior of the simulation increases neural network accuracy, we performed two-sample t-tests (p < 0.05) for each dataset to evaluate whether the average peak accuracies achieved by the feedforward and LSTM models were significantly different. The achieved accuracies and losses display the overall performance of each neural network model, whereas the relative performance of the neural network models is revealed via comparisons of their peak accuracies.

## Results

**Fig 3:**
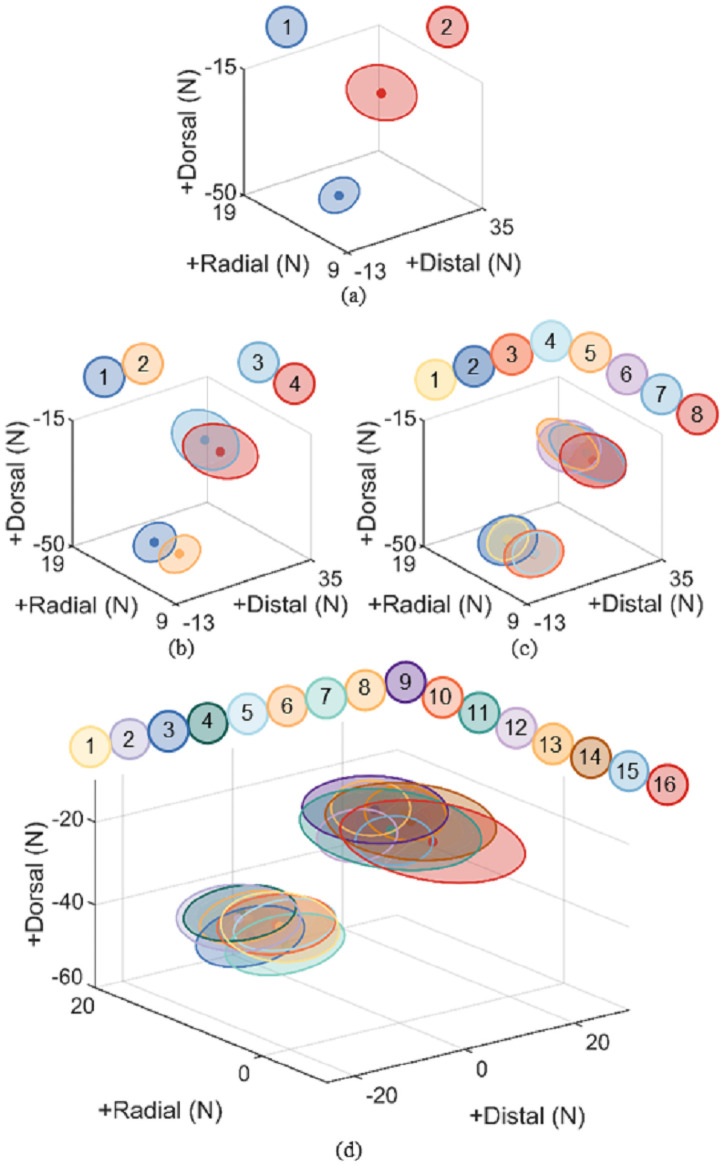
Peak thumb-tip forces across all datasets. (a)-(d) represent peak thumb-tip forces achieved in simulations from Datasets 1–4, respectively. Numbers above graphs correspond to each labeled group, which are described in Table 4. Ellipsoid centers represent mean force achieved for each labeled group. Ellipsoid radii represent 1 standard deviation from the mean. As datasets incorporate changes to more thumb muscles, the final thumb-tip forces achieved overlap more frequently across labeled groups. This increased overlap results in a more challenging classification task for each neural network model.

The simulation datasets successfully characterized the relative contributions of each muscle to the production of thumb-tip force (Fig 3). Unique muscle contributions within each dataset are illustrated by smaller percent overlap between labeled groups (Table 3). Across all datasets, two distinguishable groups consistently emerged: high FPL maximum isometric force and low FPL maximum isometric force. Despite variations in every other muscle tested, there is no overlap between any group with a high maximum isometric force of the FPL and any group with a low one (c.f., Fig 3a–3d, clear division between groups defined by distal and dorsal force direction). The percent volume overlap between thumb-tip forces generated with high versus low maximum isometric force of each other muscle analyzed was substantially higher, exceeding 35% for the APL, 41% for the ADPt, but only reaching 18.2% for the ADPo (c.f., Fig 3, division between groups defined by radial force direction).

**Table 3:** Percent volume overlap associated with groups of high or low maximum isometric force for a given thumb muscle.

Dataset	FPL	APL	ADPt	ADPo
1	0.0%			
2	0.0%	42.3%		
3	0.0%	35.8%	63.9%	
4	0.0%	59.4%	41.9%	18.2%

Each percent displayed was calculated as the volume of ellipsoid overlap (see Fig 3) for any groups of high or low maximum isometric force for a given muscle, divided by the total volume occupied by all ellipsoids in a dataset.

**Table 4:** Maximum isometric force (above or below the mean) associated with labeled groups of each dataset.

Dataset	Group Number ^a^	FPL	APL	ADPt	ADPo
1	1	High			
	2	Low			
2	1	High	High		
	2	High	Low		
	3	Low	High		
	4	Low	Low		
3	1	High	High	Low	
	2	High	High	High	
	3	High	Low	High	
	4	High	Low	Low	
	5	Low	High	High	
	6	Low	High	Low	
	7	Low	Low	High	
	8	Low	Low	Low	
4	1	High	Low	Low	Low
	2	High	High	Low	Low
	3	High	High	High	High
	4	High	High	High	Low
	5	High	Low	Low	High
	6	High	High	Low	High
	7	High	Low	High	High
	8	High	Low	High	Low
	9	Low	High	High	Low
	10	Low	Low	Low	High
	11	Low	High	Low	Low
	12	Low	High	High	High
	13	Low	High	Low	High
	14	Low	Low	High	Low
	15	Low	Low	High	High
	16	Low	Low	Low	Low

"Low" refers to a maximum isometric force below the mean (as reported in Table 1) and "High" refers to that above the mean.

**Fig 4:**
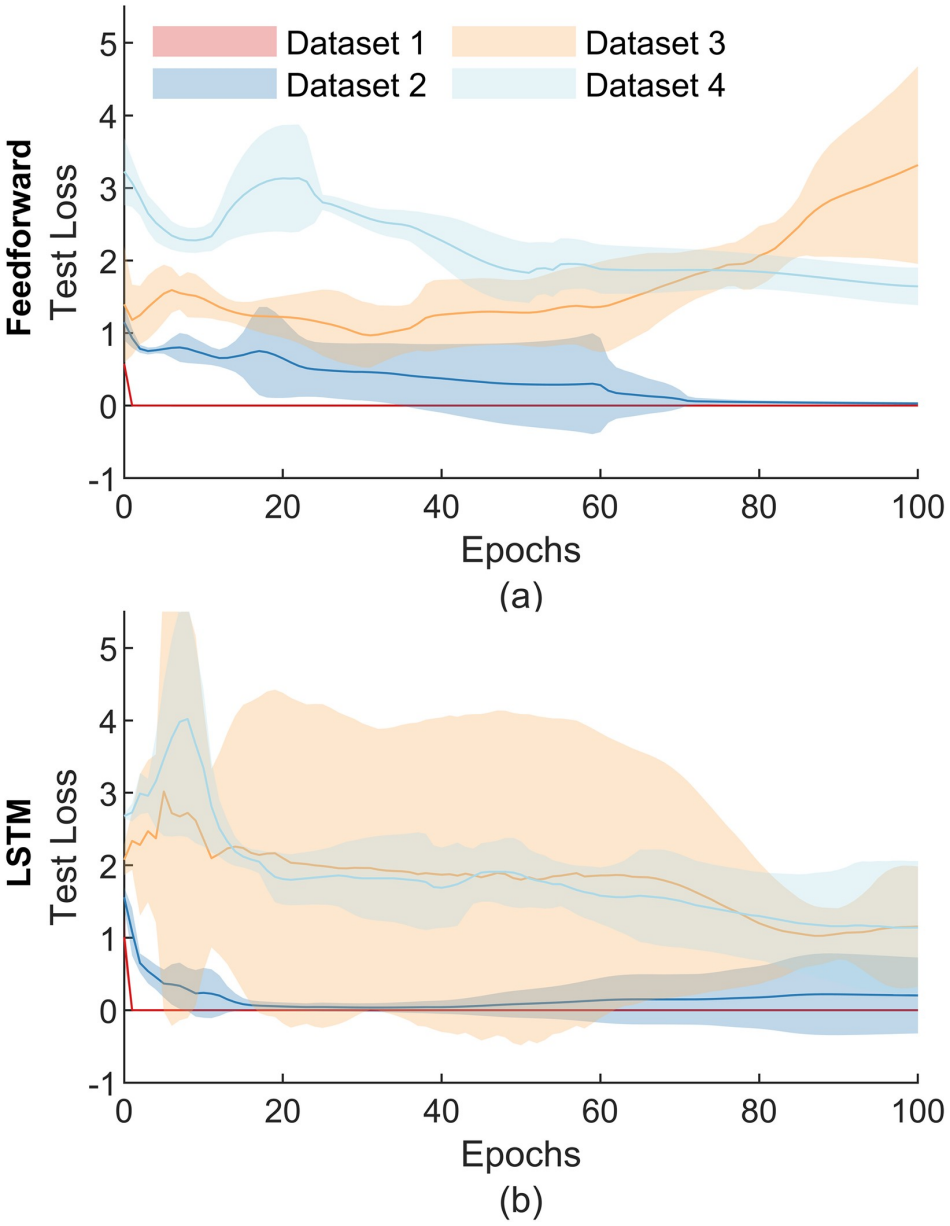
Test losses of each artificial neural network. Test losses across epochs through 5-fold cross-validation for feedforward (a) and LSTM (b) neural network models. Shaded regions represent 95% confidence intervals. Losses for datasets tended to decrease across epochs as model weights converged. Datasets which altered more muscles tended to have larger confidence intervals.

**Fig 5:**
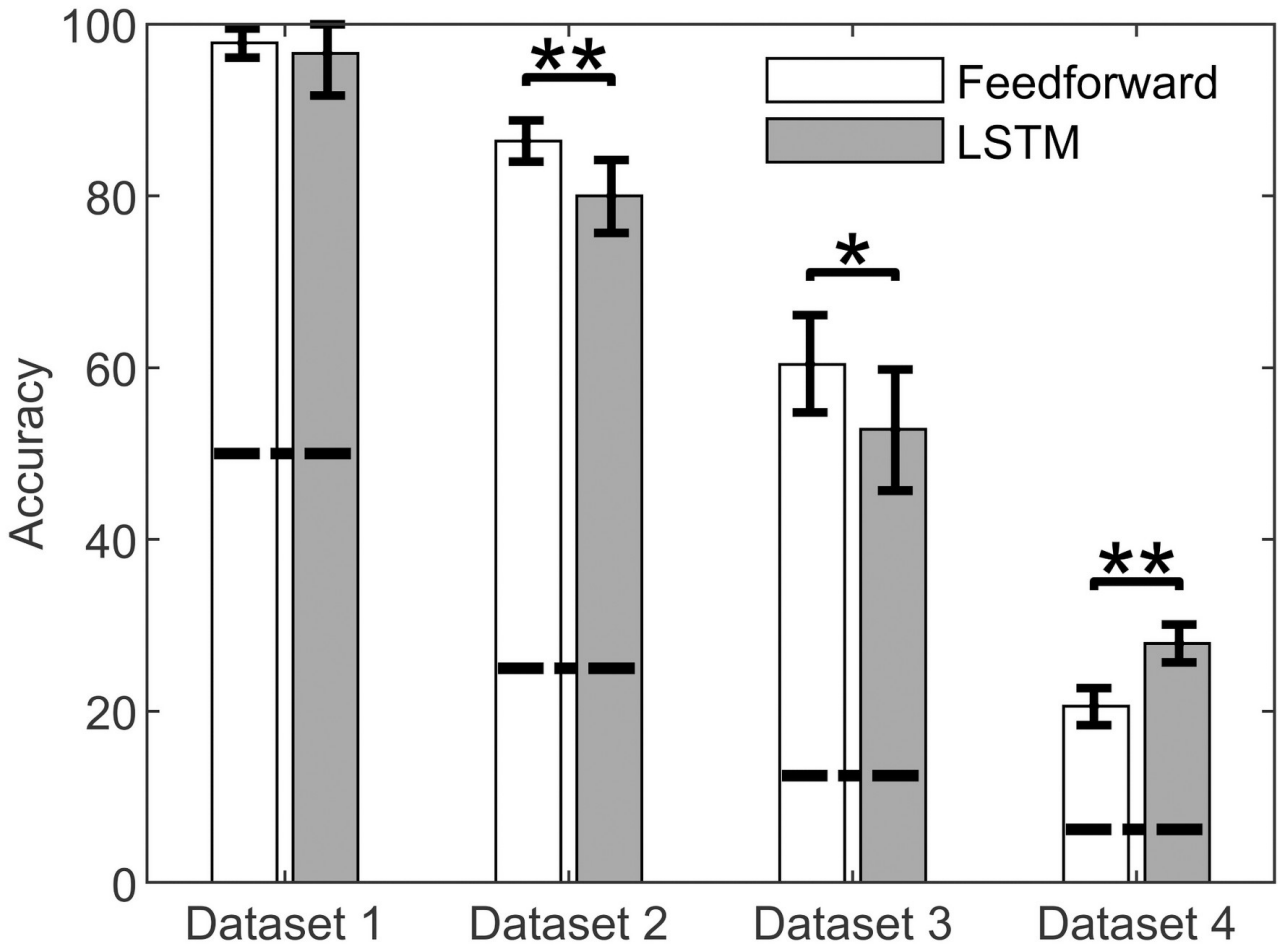
Accuracy of each artificial neural network. Average peak test accuracies achieved by both neural network models through 5-fold cross-validation. Error bars represent 95% confidence intervals and horizontal dashed lines represent the model’s accuracy for a random guess. Two-sample t-tests showed significant differences between accuracies across FF and LSTM models (* corresponds to significance p<0.05, ** corresponds to significance p<0.01).

Feedforward neural network accuracy was substantially lower and less stable in datasets with variations to more muscles. The analyses of Dataset 1 and Dataset 2 showed losses to decrease across epochs (Fig 4a) before converging at their final values. The final losses for the feedforward model were notably more consistent for simpler datasets, as shown by narrower confidence intervals across the 5-fold cross-validation process. Additionally, the peak accuracies were relatively high for Datasets 1 and 2 (Fig 5). In contrast, the peak accuracy for Dataset 1 was 97.8%, as compared to 20.5% for Dataset 4.

Similar to the feedforward model, the LSTM model performance decreased substantially for datasets that adjusted more muscles. Analyses of simpler datasets produced lower losses than those of datasets which varied additional muscles (Fig 4b). Losses across epochs varied substantially for Dataset 3 in the LSTM models, but still tended to decrease along with that of the other datasets. Again, peak accuracies substantially decreased for more complex datasets (Fig 5). The peak accuracy for Dataset 1 was 96.6%, as compared to 27.9% for Dataset 4.

Both neural network models achieved comparably high accuracies relative to random guess (Fig 5). The LSTM model trained from Dataset 4 achieved the lowest accuracies, but still reached 27.9% accuracy as compared to 6.25% for a random guess. Comparing the models via two-sample t-tests revealed the analysis of Dataset 2 and Dataset 3 to produce significantly higher peak accuracies (p<0.01 and p<0.05, respectively) using the feedforward model as compared to the LSTM model. However, the two-sample t-test also revealed the analysis of Dataset 4 to produce significantly higher peak accuracies (p<0.01) using the LSTM model as compared to the feedforward model.

## Discussion

Leveraging artificial neural networks and musculoskeletal models, we predicted changes in difficult-to-measure muscle parameters from minimal, measurable data. We specifically quantified feedforward and LSTM neural network performance in predicting the maximum isometric force of thumb muscles from simulated lateral pinch data. We report two key findings: 1. Under relatively simple conditions, neural network models can predict high and low maximum isometric force from lateral pinch data and 2. Accounting for long-term temporal dependencies in simulated lateral pinch data did not significantly improve neural network model performance. Although we did not predict explicit values of maximum isometric force in this study, accurately predicting changes in this parameter is a critical step that demonstrates the feasibility of using artificial neural networks to perform this task. With considerations for task complexity, dataset size, and Hill-type parameter of interest, the foundational work developed here could be expanded to define specific values of Hill-type parameters through categorization or regression.

The observed decrement in model performance for datasets which varied more muscle actuators may be attributable to redundancies in actuator function. The ADPt and ADPo both adduct the thumb and represent two heads of the same muscle: the *adductor pollicis*. The ADPt and ADPo have been shown to contribute to similar directional components of lateral pinch thumb-tip force in cadaveric specimens [20]. We observed this behavior in our forward dynamic simulations (Fig 3), as varying the maximum isometric force of the ADPt and ADPo produced similar changes in thumb-tip force. Thus, the similar force contributions of actuators varied in Dataset 3 and Dataset 4 made the classification task of the neural network inherently more challenging. The increased challenge to the neural network for more complex datasets is also supported by the increased overlap observed for the APL, ADPt, and ADPo in Dataset 3 and Dataset 4 (Table 3). Further investigations may leverage our simulation framework to identify the role of individual muscles in performing complex, coordinated tasks. Understanding the physical behavior of the system will be crucial to neural network model development and interpretation.

Classifying the maximum isometric force of multiple, interrelated muscles from thumb-tip force alone is challenging. The substantially higher accuracies and lower losses observed for Dataset 1 and Dataset 2 as compared to Dataset 3 and Dataset 4 may reflect a need for more diverse input variables. Datasets scaled substantially in complexity through the inclusion of changes to additional muscles. Neural network models used to classify parameters of redundant muscles may require additional types of biomechanical inputs. Artificial neural networks have been widely employed to map measured electromyographic, kinematic, and kinetic parameters [36]. These quantities may prove relevant for predicting Hill-type parameters. However, the selection of additional input variables should be completed with caution. The need for caution is illustrated by Frize et al. [37], who compared the performance of two models for making clinical decisions in intensive care units, one with 6 variables input and the other with 51 [37]. The model trained on 6 variables had a higher classification rate, lower average squared error, and converged after substantially fewer epochs. Thus, in some scenarios, inputting irrelevant parameters can impede classification performance. In this context, a key contribution of our study is that simple classifications can be performed from minimal data, such as thumb-tip force alone.

Rather than use measured thumb-tip force, we simulated dynamic thumb-tip force to input into our machine learning framework. This approach had two advantages: it enabled the efficient generation of large datasets and enabled the isolated adjustment of the maximum isometric force of the muscles we investigated. Large datasets are necessary, as a common pitfall when applying machine learning to biomechanical datasets is the *curse of dimensionality*, whereby high-dimensional models learn inadequately from too few observations in a large feature space [38]. Forward dynamic simulations can produce large numbers of observations efficiently, providing an effective means of training machine learning algorithms in the prediction of complex biomechanical movements. In the present work, this enabled the development of datasets that represented a wide physiological range of muscle parameters. This range roughly reflects an adult (approximately 29.2 y/o) male population [39] within ±2 SDs of the mean maximum isometric force for the thumb muscles analyzed. However, as the maximum isometric force remains unmeasurable *in vivo*, this is an approximation. It is also important to note that the datasets we employed did not vary in posture, likely limiting the generalizability of the reported neural network models to new postures. In the present work, this constant posture across simulations enabled the isolated variation of the maximum isometric force. Understanding to what extent changes in behavior of dynamic thumb-tip force production is attributable to the maximum isometric force enhances our fundamental understanding of thumb biomechanics, and future work could focus on expanding these methods to evaluate the effects of other variables.

The simple artificial neural network structures employed may have limited the performance achieved by each neural network model. Across each of the datasets analyzed, the feedforward and LSTM models maintained the same depth (1 layer) and width (4 hidden nodes). Maintaining the same architecture across feedforward and LSTM models enabled the fair comparison of their performance, regardless of model complexity. These models performed relatively well for Datasets 1 and 2, achieving accuracies above 96% and 80%, respectively. However, accuracies were below 30% for Dataset 4. Deeper structures may be more appropriate for complex datasets, as this would enable the model to discover more abstract features [40]. Especially in more challenging classification tasks, additional layers may enable the model to yield higher accuracies. Neural network width should also be tuned, as the inclusion of additional hidden nodes may enable quicker convergence [41]. However, use of too many hidden nodes can yield poor generalizability, which was avoided by the simple neural network structures presented. Lastly, further hyper-parameter tuning may have a substantial effect on neural network performance [42]. Despite these limitations, the neural network structure implemented in this study enabled us to test the feasibility of using artificial neural networks to predict Hill-type parameters, and to test whether feedforward or LSTM models are better suited to predict Hill-type parameters during lateral pinch. Even with relatively simple neural network models, we demonstrated that Hill-type parameters could be predicted from minimal data. We also successfully compared the performance of feedforward and LSTM neural network models. However, future works that use the described simulation and neural network frameworks to study more complex biomechanical systems may require more robust hyper-parameter tuning.

In general, the LSTM model did not perform consistently better or worse than the feedforward model (Fig 5). Notably, we observed significantly higher peak accuracies using the feedforward model as opposed to the LSTM model for Dataset 2 and Dataset 3. Yet, this result is contradicted in Dataset 4, for which the LSTM model outperformed the feedforward model. The inability of the LSTM model to consistently outperform the feedforward model may be the result of the data itself. Throughout each simulation, thumb-tip force increases at an almost constant rate before achieving its peak value. As a result, the relation between thumb-tip forces early and late in a simulation may provide little insight into underlying muscle parameters. This suggests the use of LSTM neural networks is not needed when analyzing gradually increasing lateral pinch thumb-tip force data. Rather, LSTM models are likely better suited for more dynamic tasks, such as gait analysis. For example, Kidziński et al. [43] used LSTM models in the prediction of gait events in children with healthy and pathological gait. This model achieved relatively low error (10 ms and 13 ms for foot-contact and foot-off, respectively), outperforming heuristic-based approaches. In the upper extremity, LSTM models could be suitable for studying tasks that vary substantially with time, such as predicting hand postures from electromyography [44]. In the present work, the LSTM model may have outperformed the feedforward model if the lateral pinch force varied more, such as through repetitions within each simulation.

## Conclusions

Our investigations identified artificial neural networks as a tool for approximating underlying Hill-type muscle parameters from limited lateral pinch data. Furthermore, we proposed the employment of a simulation framework to assist in the production of large datasets from which the neural network model may learn. Although accuracies calculated for all datasets were well above random guess, datasets which included muscles with similar function compromised neural network performance. Future work should test whether including other easily measurable quantities assists the classification or regression of properties for muscles of redundant function. Additionally, these investigations revealed that feedforward models, as opposed to LSTM models, may be sufficient for classifying parameters for datasets that vary only slightly over time. These investigations inform a data-driven approach to classifying Hill-type muscle parameters.
